# Geometric and electronic structures of monolayer hexagonal boron nitride with multi-vacancy

**DOI:** 10.1186/s40580-017-0107-0

**Published:** 2017-05-26

**Authors:** Do-Hyun Kim, Hag-Soo Kim, Min Woo Song, Seunghyun Lee, Sang Yun Lee

**Affiliations:** 1 School of Electrical Engineering, Korea University, 5-Ga, Anam-dong, Seongbuk-Gu, Seoul, 136-713 Republic of Korea; 2 School of Applied Chemical Engineering, Kyungpook National University, 80 Daehak-ro, Buk-gu, Daegu, 41566 Republic of Korea; 3 Department of Chemical Engineering and Materials Science, University of Suwon, Wauan-gil 17, Bongdam-eup, Hawseong-si, Gyeonggi-do 18323 Republic of Korea; 4 Fine Chemical and Material Technical Institute, Ulsan Technopark, 15 Jongga-ro, Jung-gu, Ulsan, 44412 Republic of Korea

**Keywords:** Boron nitride, Vacancy, Defect, Deformation, Band structure

## Abstract

Hexagonal boron nitride (h-BN) is an electrical insulator with a large band gap of 5 eV and a good thermal conductor of which melting point reaches about 3000 °C. Due to these properties, much attention was given to the thermal stability rather than the electrical properties of h-BN experimentally and theoretically. In this study, we report calculations that the electronic structure of monolayer h-BN can be influenced by the presence of a vacancy defect which leads to a geometric deformation in the hexagonal lattice structure. The vacancy was varied from mono- to tri-vacancy in a supercell, and different defective structures under the same vacancy density were considered in the case of an odd number of vacancies. Consequently, all cases of vacancy defects resulted in a geometric distortion in monolayer h-BN, and new energy states were created between valence and conduction band with the Fermi level shift. Notably, B atoms around vacancies attracted one another while repulsion happened between N atoms around vacancies, irrespective of vacancy density. The calculation of formation energy revealed that multi-vacancy including more B-vacancies has much lower formation energy than vacancies with more N-vacancies. This work suggests that multi-vacancy created in monolayer h-BN will have more B-vacancies and that the presence of multi-vacancy can make monolayer h-BN electrically conductive by the new energy states and the Fermi level shift.

## Background

Boron nitride (BN) is a material with a superior thermal stability [1–3] and exists in various crystal structures such as sphalerite, wurtzite, and hexagonal structures. Among them, hexagonal BN (h-BN) is a layered material which has the exactly same structure to graphite. In the case of graphite, C atoms are arranged in the hexagonal lattice structure. Similarly, B and N atoms are orderly occupied in the hexagonal structure of h-BN layers. Recently, graphene, or a layer of graphite, [4] has attracted attention due to the electrical properties such as ambipolar field effect [4], high carrier mobility [5–7], and quantum Hall effect at room temperature [8–13]. And, huge efforts have been made to tune the electronic structure of graphene theoretically [14–16] and experimentally [17–19]. Different from graphene, h-BN is electrically an insulator with a wide band gap of about 5 eV [20–24]. Due to this, less effort has been made to investigate the electrical properties of h-BN. Furthermore, converting the electrical characteristics of h-BN from an insulator to an electrical conductor is a significant and interesting task.

Several theoretical calculations have been conducted to predict the electrical properties of monolayer h-BN by structural modification. According to the calculations, the electronic structure can be affected by substituting B or N atom with different kinds of atoms [15, 21, 24, 25] or by vacancies in monolayer h-BN by removing B or N atom [20, 21, 24]. Especially, in the case of theoretical works on a vacancy in monolayer h-BN, most of the studies focus on mono-vacancy such as B- or N-vacancy rather than multi-vacancy defects. However, with the advance in etching h-BN [26, 27], creating multi-vacancy in the layered structure is not a technical barrier any more. Hence, investigating the effect of multi-vacancy on the electronic structure of h-BN layer is a significant work. Specifically, it is because h-BN is composed of two kinds of atoms different from graphene and different configurations of multi-vacancy are formed even under the same vacancy density where the number of vacancy is odd. Therefore, each vacancy configuration will have different electronic structures as well as geometric deformations. However, few efforts have been made to study a change in the electronic structure of h-BN by multi-vacancy defects.

In this study, we theoretically investigated the effect of multi-vacancy on the geometric and electronic structures of monolayer h-BN. The density of multi-vacancy was varied from mono- to tri-vacancy by removing B or N atoms from monolayer h-BN. As mentioned above, a lattice structure of h-BN with B- and N-vacancy should be considered separately in the case of mono-vacancy. Tri-vacancy is also placed in the same situation where 2B-vacancies and N-vacancy should be considered with the vacancy configuration of 2N-vacancies and B-vacancy despite the same vacancy density. Therefore, we conducted calculations on each configuration of defected hexagonal lattice of h-BN when the number of vacancy is odd (mono- and tri-vacancy). Also, the formation energy (E_form_) of h-BN with different densities of vacancy was also calculated to clarify which vacancy configuration is preferred to be formed in the structure of h-BN monolayer.

## Computational method

Our calculations based on dispersion-corrected density functional theory with the local density approximation were implemented in the PWSCF code of the QUANTUM ESPRESSO package. Perdew–Zunger parameterization and norm conserving Troullier–Martins pseudopotentials were used for the electron exchange–correlation interaction. Also, spin-polarization was considered in all cases of the calculations. We employed a 5 × 5 supercell structure of monolayer h-BN which contains 50 atoms. And, a defected structure was realized by removing B and N atoms according to the number of a vacancy. Triclinic supercell of 12.5 × 12.5 Å with periodic boundary conditions was used to simulate pristine and defected monolayer h-BN. A vacuum region of 15 Å was added to avoid the interactions between periodic images. The equilibrium atomic position was determined by relaxing all B and N atoms in pristine and defected structures. The Brillouin zone of the supercell was sampled by a 12 × 12 × 1 Monkhorst–Pack grid. A plane wave basis set with a kinetic cutoff energy of 30 Ry was used.

**Fig. 1:**
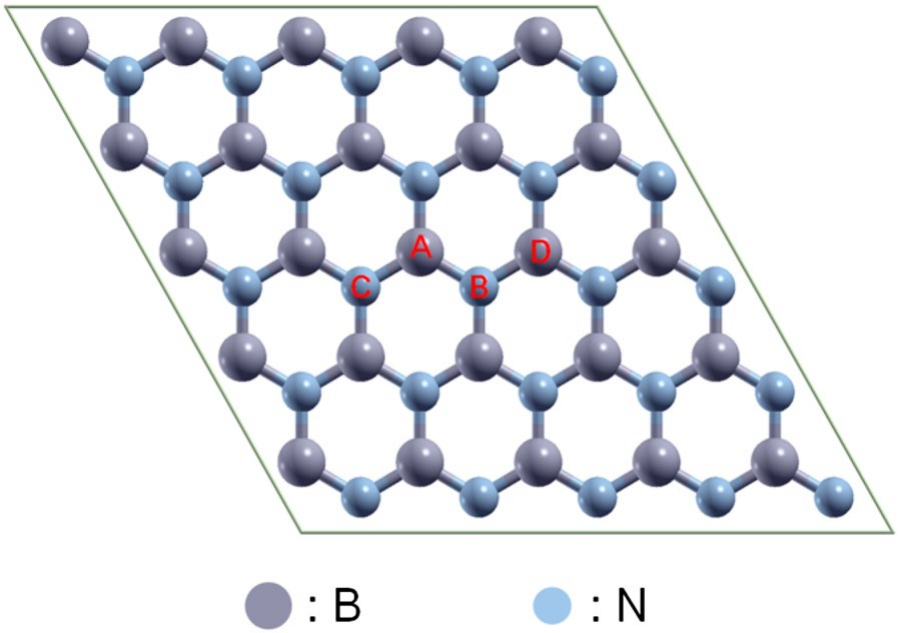
Schematic illustration of multi-vacancy creation on the 5 × 5 monolayer h-BN supercell with 50 atoms. *A*, *B*, *C*, and *D* present a vacancy site corresponding to mono-(1V_B_ and 1V_N_), di-(2V_BN_), and tri-(3V_2NB_ and 3V_2BN_) vacancy

The defective h-BN was realized by removing B or N atom one by one from pristine h-BN structure, depending on the number of vacancy. Figure 1 presents the schematic illustration of modeling h-BN with mono-, di-, and tri-vacancy. In the case of mono-vacancy, two cases were considered by eliminating the B or N atom in the position of A or B (hereafter, 1V_B_ and 1V_N_), respectively. B and N atoms corresponding to A and B sites were removed at the same time to model monolayer h-BN with di-vacancy (hereafter, 2V_BN_). Two defective configurations of tri-vacancy were realized by removing atoms in the position of C–A–B and A–B–D (hereafter, 3V_2NB_ and 3V_2BN_), respectively.

The formation energy of each structure was calculated by using the following equation:

$$E_{form} = E_{V} - \frac{{n - n_{V} }}{n}E$$

where, E_V_ and E are the total energy of defective and pristine h-BN, respectively. And, n and n_V_ are the number of atoms in pristine structure and vacancies in defective h-BN.

## Results and discussion

### Mono-vacancy

**Fig. 2:**
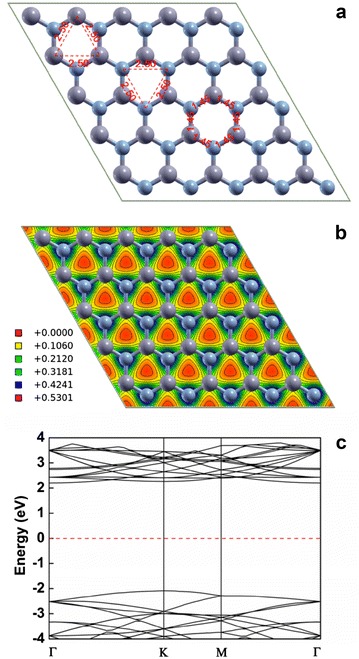
**a** Relaxed structure, **b** isodensity plot, and **c** electronic band structure of pristine h-BN. The Fermi level is set to be zero

Before investigating the geometric and electronic structures of monolayer h-BN with mono-vacancy, it is necessary to study pristine h-BN in order to distinguish geometric and electronic differences between pristine and defective h-BN. Thus, the geometric and electronic structures of pristine lattice structure were calculated. Figure 2 shows a relaxed structure, isodensity plot, and electronic band structure of pristine h-BN. As shown in Fig. 2a, the bonding length between B and N atoms was calculated to be 1.45 Å from the relaxed structure. As marked with a triangle in the figure, the distances between B atoms and N atoms are all of the same length (2.50 Å). The isodensity plot in Fig. 2b shows that when a vacancy does not exist in monolayer h-BN, electron density is evenly distributed throughout the supercell. Figure 2c displays the electronic band structure of pristine h-BN which was calculated along the Γ-K-M-Γ path in the Brillouin zone. The band structure reveals that pristine h-BN has an indirect band gap of 4.3 eV in the electronic structure, which is consistent with previous calculations [25, 28].

**Fig. 3:**
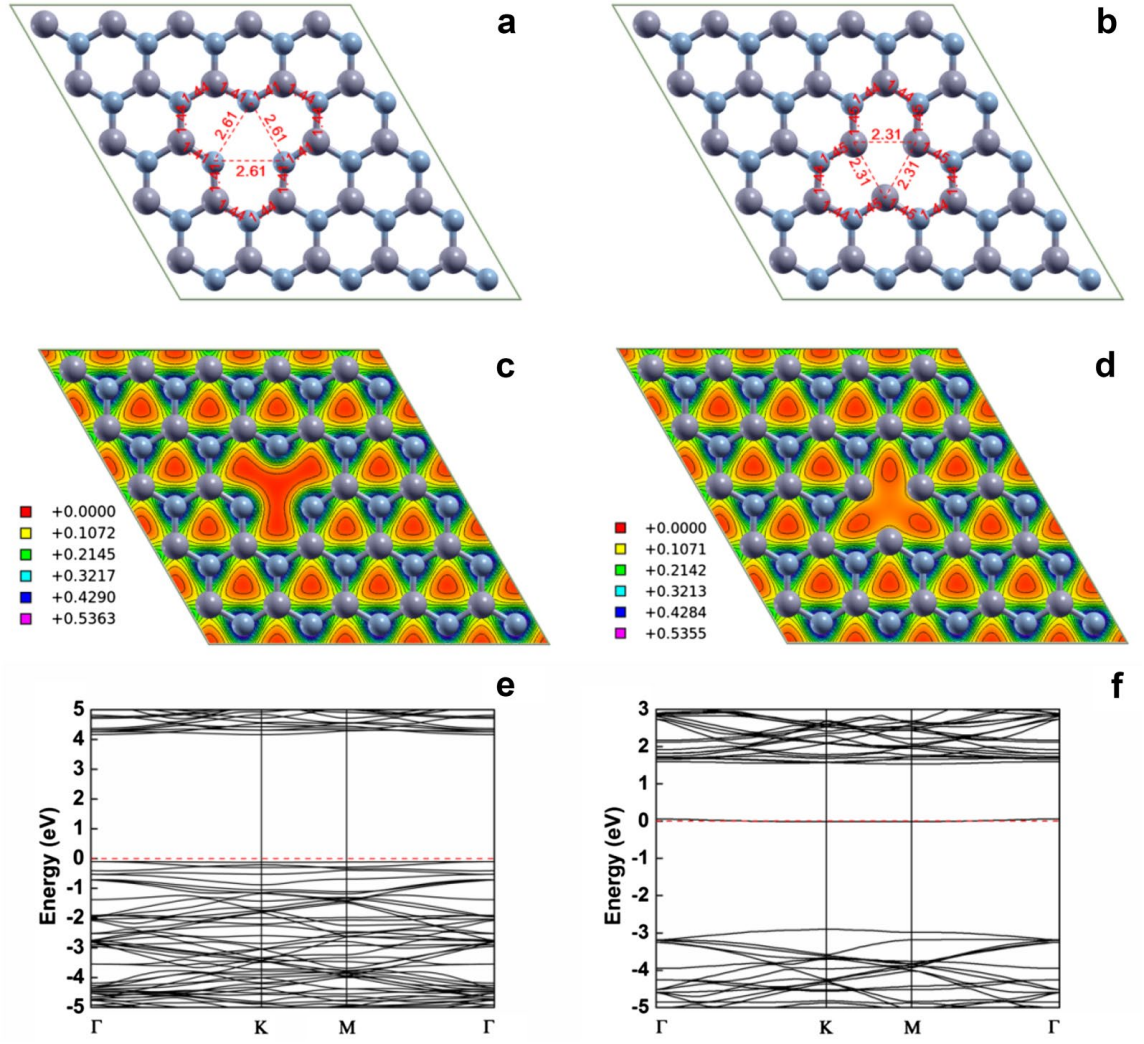
The *left column* shows **a** relaxed structure, **b** isodensity plot, and **c** electronic band structure of h-BN with 1V_B_, and the* right column* displays **b** relaxed structure, **d** isodensity plot, and **f** electronic band structure of h-BN with 1V_N_. The Fermi level is set to be zero

Different from graphene, h-BN can have two kinds of defective structures in the case of mono-vacancy (1V_B_ and 1V_N_), depending on a vacancy element. Thus, both different configurations should be separately considered to study the effect of mono-vacancy on the geometric and electronic band structures. Figure 3a and b display relaxed supercell structures with B and N vacancy, respectively. To evaluate the degree of distortion, the distance between atoms around the vacancy site was measured in each case. For this, the atoms around mono-vacancy were connected by lines which lead to a triangle. When 1V_B_ is created in the structure as seen in Fig. 3a, all distances between the atoms are measured to be 2.61 Å which is greater than 2.50 Å measured from pristine h-BN. The relaxed structure of the defected h-BN and the measured distance are consistent with the results reported by previous works [21, 24]. The elongated distance indicates that the presence of mono-vacancy distorted a geometric structure of monolayer h-BN. And, the bonding lengths between the atoms at the edge site around B vacancy are changed to 1.41 and 1.44 Å. This also means that a geometric distortion occurred by B-vacancy in comparison with pristine lattice structure. Any planar distortion perpendicular to the monolayer did not happen after relaxation. In the case of 1V_N_, a similar shape of deformation to B-vacancy appears in the relaxed structure, and the distance between B atoms is measured to be 2.31 Å. This relaxed structure and the distance are also consistent with the previous works [21, 24]. The bonding lengths of atoms existing at the edge of mono-vacancy ranges from 1.44 to 1.45 Å, indicating that the presence of N-vacancy causes a geometric distortion in the supercell. Also, there was no planar deformation to the vertical axis of h-BN lattice structure. Interestingly, the distance between B atoms in Fig. 3b is shorter than the length between N atoms in Fig. 3a. This reason can be found in the isodensity plot displayed in Fig. 3c and d. When B atom is missing in the supercell, the repulsion between N atoms around the vacancy breaks out as shown in Fig. 3c. Meanwhile, B atoms around N-vacancy attract one another, resulting in a shortened distance of 2.31 Å and an elongated bonding length of 1.45 Å. Therefore, the shortened distance can be attributed to the attraction between B atoms around N-vacancy. This different situation gives rise to different electronic structures as shown in Fig. 3e and f. In the case of 1V_B_, a gap in the band structure has a similar value to that of pristine monolayer h-BN. However, the presence of 1V_B_ shifts the Fermi level downward valence band. In the case of 1V_N_, a new energy state is created along the Fermi level as shown in Fig. 3f, which might help electron jump to conduction band.

### Di-vacancy

**Fig. 4:**
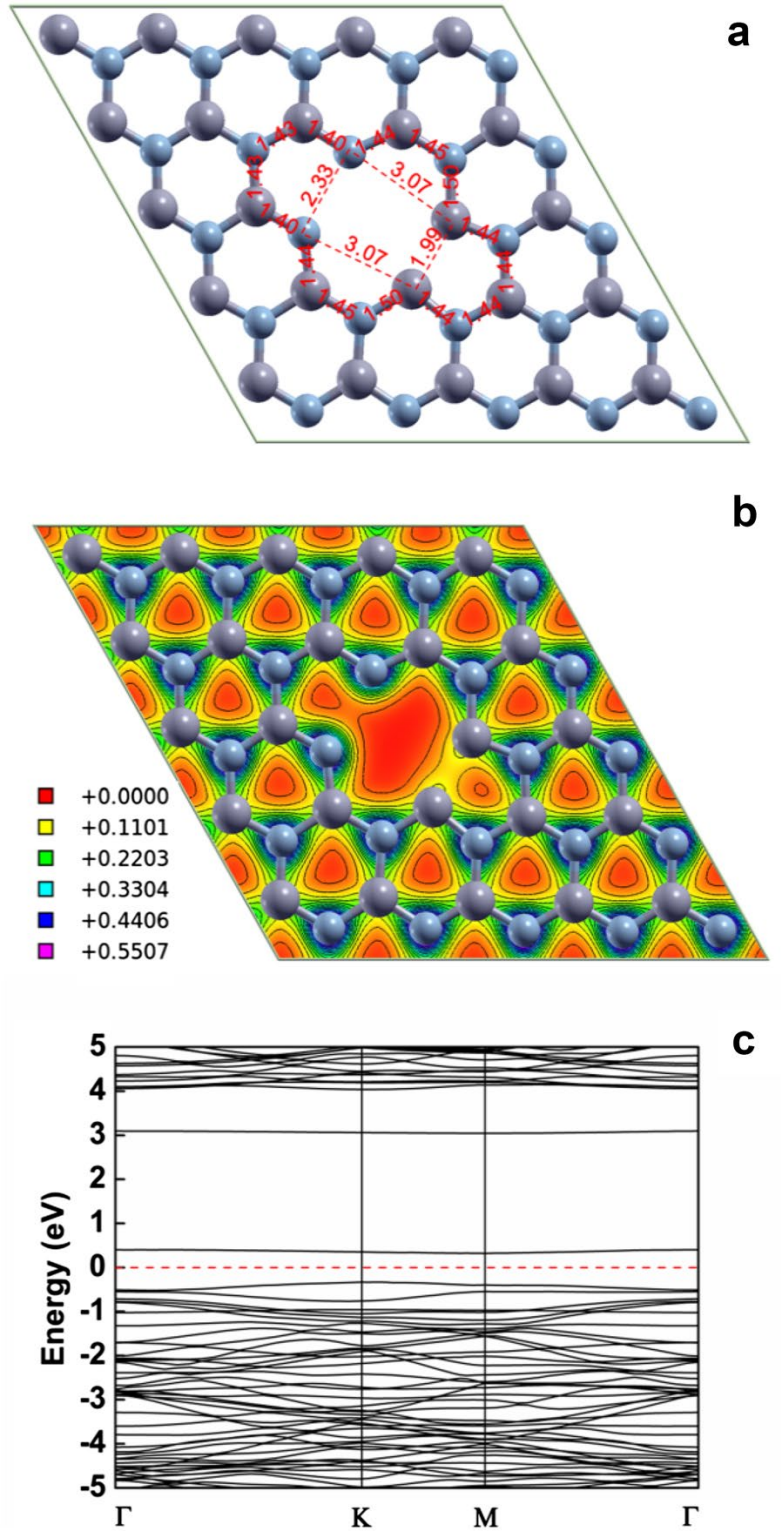
**a** Relaxed structure, **b** isodensity plot, and **c** electronic band structure of h-BN with 2V_BN_. The Fermi level is set to be zero

Different from an odd-number vacancy, di-vacancy in h-BN has only one defective configuration which includes all elements. B and N atoms positioned at A and B site in Fig. 1 were removed to model di-vacancy in the supercell. Figure 4a displays a relaxed structure of monolayer h-BN with 2V_BN_. To examine the degree of geometric distortion, the distance between atoms at the edge site around the vacancies was measured as marked with lines shown in Fig. 4a. As a result, the distance between B atoms around 2V_BN_ was measured to be 1.99 Å while the distance between N atoms at the edge was calculated to be 2.33 Å. These values are less than the distance calculated in pristine lattice structure. In the case of the distance between B and N atoms around 2V_BN_, the length was calculated to be 3.07 Å in all cases as seen in Fig. 4a. It can be understood that this is due to the repulsion by the localized electrons around N atoms and the attraction between B atoms, resulting in an elongated distance of 3.07 Å greater than 2.50 Å. No planar distortion was observed toward the direction perpendicular to the monolayer. Also, the di-vacancy influences on the bonding length between atoms at the edge around the vacancies, where the bonding length is distributed from 1.40 to 1.5 Å. Consequently, this leads to a distortion in the geometric structure, and changes the electron density distribution as presented in Fig. 4b. The distribution also shows that a weak covalent bond is formed between B atoms due to the relatively high electron density. This apparently explains why the distance between B atoms was shortened to 1.99 Å in monolayer h-BN with 2V_BN_. The structural change and the presence of di-vacancy lead to an alteration in the electronic structure. As displayed in Fig. 4c, two new energy states, which do not show in the band structure of pristine h-BN, appear between the valence and conduction band. The new energy states can contribute to electron jumps to conduction band.

### Tri-vacancy

**Fig. 5:**
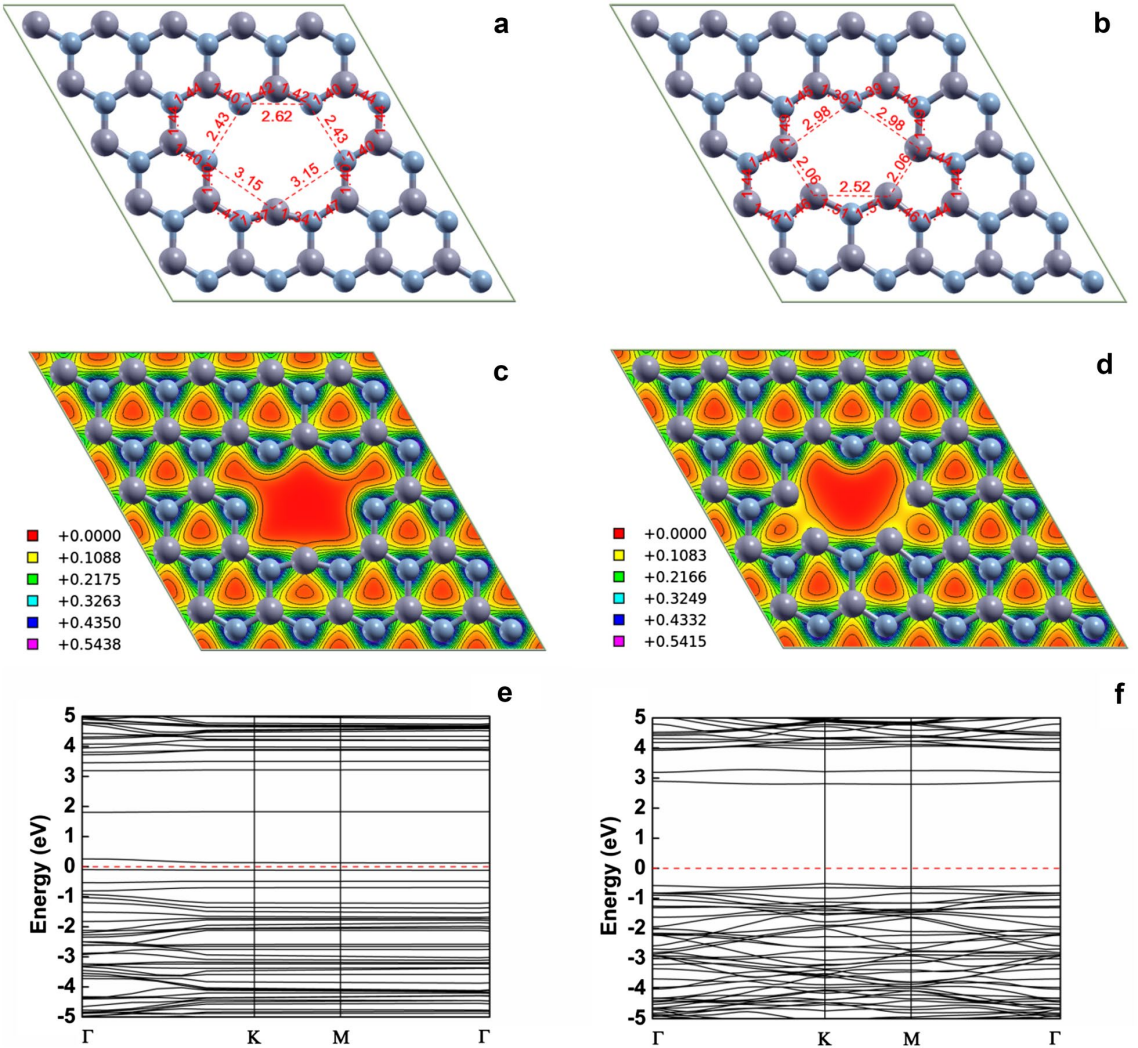
The *left column* shows **a** relaxed structure, **b** isodensity plot, and **c** electronic band structure of h-BN with 3V_2BN_, and the* right column* displays **b** relaxed structure, **d** isodensity plot, and **f** electronic band structure of h-BN with 3V_2NB_. The Fermi level is set to be zero

Similar to mono-vacancy, monolayer h-BN with tri-vacancy is present in the form of two defective configurations such as 3V_2BN_ and 3V_2NB_. Figure 5a shows the relaxed hexagonal lattice structure of monolayer h-BN with 3V_2BN_. To investigate the degree of distortion by the vacancies, the atoms at the edge around the vacancies were connected by lines. As seen in the figure, the lines form a pentagon and have different lengths of 2.43, 2.62, and 3.15 Å. The different distances indicate that the structure was deformed by the presence of 3V_2BN_ in comparison with the pristine structure. This is also supported that the bonding lengths between atoms at the edge sites range from 1.34 to 1.47 Å, which are deviated from the value of 1.45 Å calculated from pristine h-BN. Figure 5b displays a deformed structure of monolayer h-BN by 3V_2NB_ where the atoms at the edge sites are also connected by lines. The sides of pentagon have different values of 2.06, 2.52, and 2.98 Å, and the bonding lengths of atoms at the edge are in the range between 1.38 and 1.51 Å. As seen in mono- and di-vacancy, the distance between B atoms around the vacancies becomes shorter than 2.50 Å corresponding to the distance between B atoms in pristine h-BN. The isodensity plot shown in Fig. 5c and d can explain the reason why the distance between B atoms became short by the vacancies. In the case of 3V_2BN_, N atoms around the vacancies in Fig. 5c have much higher electron density than B atoms while almost few electrons exist around B atoms at the edge sites in Fig. 5c. However, in the case of 3V_2NB_, B atoms at the edge site have more electron density and form a weak covalent bond by attraction between two B atoms as seen in Fig. 5d. Consequently, these different electron densities in the lattice structure lead to different electronic structures. As displayed in Fig. 5e and f, new energy states are generated between valence and conduction band in both cases of 3V_2BN_ and 3V_2NB_. However, in the case of 3V_2BN_, the new states are evenly distributed in the gap defined by valence and conduction band, and the Fermi level exists between the states. Meanwhile, in the case of 3V_2NB_, the new states are relatively unevenly positioned, and the Fermi level exists away from the new states, compared with the electronic structure created by 3V_2BN_. Nevertheless, it can be expected that the new energy states can help electrons jump from valence to conduction band, converting monolayer h-BN to an electrically conductive material.

### E_form_ of the vacancies

**Fig. 6:**
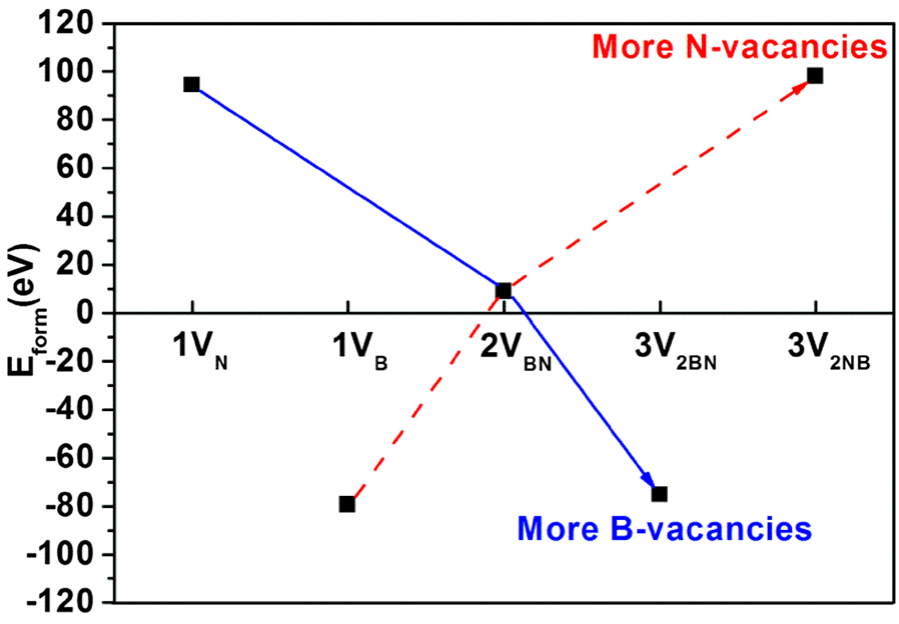
Plot of E_form_ with respect to vacancy density. Monolayer h-BN with more B vacancies is energetically preferable to be formed even under the same vacancy density

It has been revealed that defective h-BN structures have different geometric deformations and electronic structures even under the same vacancy density. This means that each structure has unique E_form_, depending on the configuration and the number of a vacancy defect. Therefore, calculating E_form_ of each defective structure can give information about which vacancy is an energetically preferable structure. Figure 6 exhibits a plot of E_form_ with respect to the number of a vacancy. In the case of mono-vacancy, E_form_ of 1V_B_ has much lower value than that of 1V_N_, meaning that B-vacancy is an energetically preferable defect rather than N-vacancy. The E_form_ of 2V_BN_ has a positive value of 8.9 eV, suggesting that di-vacancy is not preferable to be energetically formed. A similar trend seen in mono-vacancy is found in the case of tri-vacancy. While E_form_ of 3V_2BN_ has a negative value of −75.2 eV, 3V_2NB_ has a positive value of 98.1 eV. This means that tri-vacancy is energetically preferable to be present in the configuration of 3V_2BN_ rather than of 3V_2NB_. From this result, it can be said that when h-BN has the same or greater number of B-vacancy than that of N-vacancy out of the total number of vacancies, the defective h-BN structure is energetically preferable to be formed. Therefore, it is predicted that multi-vacancy created in the monolayer h-BN will have more B-vacancies than N-vacancies.

## Conclusions

In this work, we theoretically investigated the effect of multi-vacancy on the geometric deformation and the electronic structure of monolayer h-BN. The presence of vacancy resulted in a geometric deformation which leads to a change in the electronic structure of monolayer h-BN irrespective of the number of a vacancy. However, a planar deformation did not break out at any case in the relaxed structure. Specifically, regardless of vacancy density, the repulsion between N atoms around vacancies appeared due to delocalized electrons. And, attraction occurred between B atoms at the edge of vacancies. The presence of vacancies and the geometric deformation in the hexagonal lattice structure created new energy states in the band gap and shifted the Fermi level. Such a change by vacancies can help electrons jump from valence to conduction band, opening a possibility to convert h-BN into an electrical conductor.

The calculation of E_form_ revealed that multi-vacancy with more B vacancies is energetically preferable to be formed even under the same vacancy density. The result suggests that monolayer h-BN will have more B-vacancies than N-vacancies when multi-vacancy is created in the hexagonal lattice structure.

## Abbreviations

BN: boron nitride
h-BN: hexagonal boron nitride
E_form_: formation energy
